# Antimicrobial Resistance Pattern of ***Shigella*** species Over Five Years at a Tertiary-care Teaching Hospital in North India

**DOI:** 10.3329/jhpn.v29i3.7878

**Published:** 2011-06

**Authors:** Urvashi, Sonal Saxena, Renu Dutta

**Affiliations:** Department of Microbiology, Lady Hardinge Medical College and Associated Hospitals, New Delhi, India

Sir,

Shigellosis is a global human health problem, especially in developing countries where there are inadequate hygiene and unsafe water supplies (1,2). *Shigella* accounts for a significant proportion of cases of bacillary dysentery in many tropical and subtropical countries (3,4). *Shigella* is one of the most important causes of gastroenteritis-induced deaths in 3-5 million children aged less than five years in developing countries (5). The emergence of multiple drug resistance to cost-effective antimicrobials against *Shigella* is a matter of concern in developing countries (6). It is also well-documented that pandemic strains often exhibit multiple antimicrobial resistance and induce severe illness with high case fatality in all age-groups (7). In the tropics, most infections occur due to *S. flexneri* and infections that occur primarily due to *Shigella sonnei* are less common (8,9).

Over a decade ago, results of active surveillance studies showed that, in most endemic countries, especially in Asia and sub-Saharan Africa, there was an emergence of multidrug resistance to different antimicrobials, including ampicillin, co-trimoxazole, and nalidixic acid (9).

Besides the temporal changes in the antibiogram of *Shigella* species, it is well-known that antimicrobial susceptibility patterns in *Shigella* may differ between geographical areas. Such differences are never stable and may change rapidly, especially in places where antimicrobials are used excessively, particularly in developing countries (1). This warrants for robust monitoring of the pattern of antibiogram for this organism.

Hence, the present study was conducted to determine the incidence and prevalence of *Shigella* serogroups isolated from cases of dysentery during a five-year period from 2004 to 2008 and also to determine the drug resistance pattern of *Shigella* in a tertiary-care hospital in North India.

In this cross-sectional study, patients presenting with acute, bloody diarrhoea, suggestive of shigellosis, during 2004-2008, were included. Stool samples were collected from all patients presenting with symptoms of diarrhoea, blood or mucus in stool, fever, and abdominal pain. In total, 12,983 stool samples were received for culture and routine microscopy during 2004-2008. All the samples were subjected to routine microscopy and were examined for pus cells, trophozoites, and cysts. Only those samples which showed pus cells and/or red blood cells were cultured. The stool specimens from such symptomatic patients were cultured on differential and selective media, including MacConkey agar, Xylose-lysine desoxycholate agar, and deoxycholate citrate agar as primary plates. Loop-full sample was also inoculated into selenite-F broth. The plates were incubated at 37 ^o^C for 24 hours. Suspected colonies on solid plates, which were Gram-negative upon Gram-staining and oxidase test-negative, were biochemically characterized as per standard procedures. Presumptive *Shigella* isolates on Triple Sugar Iron agar were serotyped by slide agglutination tests with *Shigella* polyvalent grouping and monovalent antisera (Denka Seiken Co. Ltd., Tokyo, Japan).

Antimicrobial susceptibility testing and resistance patterns of the *Shigella* isolates to various antimicrobials were determined by the disc-diffusion technique (10). Every inoculum was prepared by inoculating 5 mL of Mueller-Hinton broth with five colonies of an 18-hour old pure *Shigella* culture, followed by incubation in ambient air and at 37 ºC for 16 hours. Turbidity of the broth was equilibrated to match with 0.5 McFarland standards. A sterile cotton swab was dipped into the standardized suspension, drained, and used for inoculating 25 mL of Mueller-Hinton agar in a 90-mm plate. The inoculating plates were air-dried, and antimicrobial discs, included ampicillin (10 μg), chloramphenicol (30 μg), co-trimoxazole (25 μg), tetracycline (30 μg), gentamicin (10 μg), nalidixic acid (30 μg), and ciprofloxacin (5 μg). Furazolidone [Oxoid (UK) and Hi-Media (Mumbai, India)] were placed on them. The plates were inverted and incubated in ambient air at 37 ºC for 18 hours. Zone-sizes were measured and compared with those of *Escherichia coli* ATCC25922 which served as a control strain.

Minimum inhibitory concentrations were determined against ceftriaxone and cefotaxime using broth dilution techniques as per the guidelines of the Clinical Laboratory Standards Institute (10,11). MIC_50_ and MIC_90_ were calculated using cumulative frequency table.

In total, 12,983 stool samples were received for culture and routine microscopy during 2004-2008. Of these samples, 2,109 (16.2%) were from indoor patients while the remaining samples were from outdoor patients. Upon performing routine microscopy, only those samples which showed pus cells and/or red blood cells were cultured. In total, 4,900 such samples were cultured. *Shigella* species were isolated from 106 such patients—74 (69.8%) males and 32 (30.1%) females—during 2004-2008. The age distribution of patients who were positive for *Shigella* ranged from 23 days to 72 years, with a mean age of 17.66 years. The most commonly-affected age-group was 1-5 years (39.6%), followed by the age-group of less than one year (27.3%). All the four serogroups were isolated with the following distribution: 73 strains of *S. flexneri* (68.8%), 26 strains of *S. dysenteriae* (24.5%), six strains of *S. boydii* (5.66%), and one strain of *S. sonnei* (0.9%). Resistance patterns against the applied antimicrobials were as follows: nalidixic acid–94.3%; ampicillin–88.6%; trimethoprim-sulphamethoxazole–84.9%, tetracycline–79.2%; ciprofloxacin–67.9%; chloramphenicol–54%; and gentamicin–17.9% (Table 1).

**Table 1.** Antimicrobial drug resistance in *Shigella*

Antimicrobial drug	Resistant isolates		Serogroups							
			*S. flexneri* (n=73)		*S. dysenteriae* (n=26)		*S. boydii* (n=6)		*S. sonnei* (n=1)	
	No.	%	No.	%	No.	%	No.	%	No.	%
Ampicillin	94	88.6	70	95.8	20	33.3	3	50	1	100
Co-trimoxazole	90	84.9	69	94.5	18	69.2	2	33.3	1	100
Tetracycline	84	79.2	58	79.4	21	80.7	4	66.6	1	100
Chloramphenicol	58	54	46	63.3	12	46.1	0	00	0	00
Gentamicin	19	17.9	13	17.8	6	23	0	00	0	00
Nalidixic acid	100 94.3	69	94.5	26	100	4	66.6	1	100
Ciprofloxacin	72	67.9	44	60.2	15	57.6	0	00	0	00
Furazolidone	4	3.77	4	5.4	0	00	0	00	0	00
Cefotaxime	19	17.9	12	16.4	6	23	1	16	0	00
Ceftriaxone	13	12.2	10	13.6	3	11.5	0	00	0	00

Twelve distinct resistance patterns were encountered in all the *Shigella* strains tested (Table 2). One hundred four isolates (98.11%) were multiple drug-resistant (MDR). Twelve strains were resistant to seven of the eight antimicrobials tested. Sixty-nine strains were resistant to six of the eight antimicrobials.

**Table 2.** Multiple drug resistance patterns amo-ng *Shigella* species

Antimicrobials resistance pattern	No. of isolates
R to 10 antimicrobials	
A,T,C,Co,Cf,G,Na,Fx,Ce,Ci	3
R to 9 antimicrobials	
A,T,C,Co,Cf,G,Na,Ce,Ci	1
R to 8 antimicrobials	
A,T,C,Co,Cf,G,Na,Fx,	1
A,T,C,Co,Cf,G,Na,Ci	3
A,T,C,Co,Cf,G,Na,Ce	2
R to 7 antimicrobials	
A,T,C,Co,Cf,Na,Fx	2
A,T,C,Co,Cf,Na,G	5
A,T,C,CO,Ce,Ci,G	8
A,T,Co,Cf,Ci,Na,Fx	4
R to 6 antimicrobials	
A,C,C0,T,Cf,Na	50
A,C,Co,T,Cf,Ce	2
A,C,CO,T,Cf,Ci	2
A,C,Co,Cf,G,T	7
R to 5 antimicrobials	
T,C,Co,Cf,Na	6
A,T,C,Co,Cf	4
A,T,C,Cf,Na	1
R to 4 antimicrobials	
A,C,Cf,T	1
R to 3 antimicrobials	
C,Co,Cf	1
A,C,Co	1
Total	104

A=Ampicillin;

C=Chloramphenicol;

Ce=Cef-triaxone;

Cf=Ciprofloxacin;

Ci=Cefotaxime;

Co=Co-trimoxazole;

Fu=Furazolidone;

G=Gent-amicin;

Na=Nalidixic acid;

T=Tetracycline

MIC_90_ was calculated at 32 µg/mL while MIC_50_ was found to be 16 µg/mL for both cefotaxime and ceftriaxone. Shigellosis occurs both in epidemic and endemic forms in children and is a major public-health problem in developing countries. This retrospective study demonstrated a high level of antimicrobial resistance pattern in *Shigella* species isolated from stool samples over a five-year period at a tertiary-care teaching hospital in Delhi, north India.

The majority (67%) of the *Shigella* species were isolated from children aged less than five years, which is similar to findings of other studies (4,8). The fact that the majority of *Shigella* isolates are from the paediatric population is reported in the literature where 70% of all infections occur in children aged less than 15 years (1).

The frequency of different species of *Shigella* varies in different countries. The most abundant species of *Shigella* in the present study was *S. flexneri* which is similar to studies in Zahedan, Iran (2,3), Ghana (4), and western Nepal (5). A multicentre study by Seilden *et al.* also found *S. flexneri* as the commonest species in all, except Thailand where *S. sonnei* was the common isolate (12). A significant longitudinal transition of species from *S. flexneri* to *S. sonnei* and change in antimicrobial resistance pattern have been observed in Viet Nam by Vinh *et al.* (13). However, in our setting, *S. flexneri* continues to predominate.

Over the past decades, *Shigella* spp. have become progressively more resistant to most widely-used and inexpensive antimicrobials. The last two decades have seen an emergence of multidrug-resistant (MDR) strains of *Shigella* spp. The World Health Organization currently recommends ciprofloxacin (or other fluoroquinolones) as the drug of choice for therapy of *Shigella* infections in both adults and children. In addition, ceftriaxone, pivmecillinam (amdinocillin pivoxil), and azithromycin are also considered alternative drugs suitable for the treatment of *Shigella*-associated disease (14). However, strains of *Shigella* spp. that are resistant to ciprofloxacin have been described in Asia (13,15). Also, strains of *Shigella* spp. producing extended-spectrum β-lactamases (ESBL) conferring resistance to third-generation cephalosporins have also been reported (16).

*S. flexneri,* the commonest isolate in our study, showed a high degree of resistance to most commonly-used drugs, such as nalidixic acid (94.3%), ampicillin (95.8%), co-trimoxazole (94.5%), tetracycline (79.4%), ciprofloxacin (60.2%), and chloramphenicol (63.3%).

In this study, multiple drug resistance to as many as seven antimicrobials was observed. Similar findings were reported in other studies from other geographic areas, such as from Ethiopia and recently from Viet Nam (1,13).

Most *S. flexneri* isolates in our study were susceptible to gentamicin and furazolidone which support the findings of the study conducted by Taneja *et al.* (17). However, the relative increase in the resistance to ciprofloxacin (67.2%) in the study by Neogi in all *Shigella* spp., compared to other studies (2,3,5-7,16), indicates that there is a great need to reduce the indiscriminate use of potent antimicrobials, such as ciprofloxacin (15).

Some degree of regulation of antimicrobial use is necessary as most antimicrobials are available over-the-counter and can be bought without any prescription. There is a need to educate both general public and health practitioners that most cases of diarrhoea do not require antimicrobials. Periodic monitoring of drug resistance in enteropathogens should be carried out in different geographic areas so that an appropriate agent can be chosen for empiric therapy. Currently, there are effective optional therapies, and ceftriaxone is used as an alternative when the patient does not respond to treatment with fluoroquinolone. A marked increase in resistance to nalidixic acid and newer fluoroquinolones, combined with ESBL-mediated third-generation cephalosporin resistance, would leave limited treatment options for those with life-threatening bacterial infections.
